# Microstructural and Thermo-Mechanical Characterization of Furfurylated Douglas Fir

**DOI:** 10.3390/polym14214641

**Published:** 2022-10-31

**Authors:** Xuefei Jiang, Jing Wang, Ziheng Wang, Feiyue Hua, Sheng He, Buyun Lu, Xiang Wang, Xuefeng Zhang, Weiqi Leng

**Affiliations:** 1 Jiangsu Co-Innovation Center of Efficient Processing and Utilization of Forest Resources, Nanjing Forestry University, Nanjing 210037, China; 2 China National Bamboo Research Center, Hangzhou 310012, China; 3 Center of Chemistry for Frontier Technologies, Department of Chemistry, Zhejiang University, Hangzhou 310027, China; 4 Department of Sustainable Bioproducts, Mississippi State University, Starkville, MS 39762, USA

**Keywords:** wood furfurylation, visualization, thermodynamic properties

## Abstract

Fast-growing wood has become a major source of materials for the wood industry in recent years, but defects have limited its use. Therefore, modification is urgently needed for the more efficient application of wood products. In this study, a 30 to 50% solution of furfuryl alcohol (FA) was impregnated into Douglas fir sapwood. The microstructure and thermal properties of the specimens before and after furfurylation were evaluated by different techniques. The weight percentage gain (WPG) of modified wood increased up to 22.97%, with the polymerized FA distributed in cell lumens and cell walls, as well as chemically bound to wood components. The polyfurfuryl alcohol (PFA) was mainly located in the tracheids, ray parenchyma cells, and resin canals. In addition, the furfurylated cell walls were greatly thickened. Raman spectra showed that modified wood had significant background fluorescence that covered other peaks. Differential Scanning Calorimetry analysis revealed that the cross-linking reaction between FA and wood changed the shape of curves, with no endothermic or exothermic peaks within the programmed temperature. Moreover, Thermogravimetry and Dynamic Mechanical Analysis results both confirmed that the furfurylation increased the thermal stability of Douglas fir. The percentage of the final mass loss of untreated specimen was 80.11%, while the highest one of furfurylated specimen was 78.15%, and it gradually decreased with increasing FA concentration. The storage modulus (E′) and loss modulus (E″) of the furfurylated wood were both lower, and the damping factor (tan δ) was higher than the untreated one. When the temperature reaches about 75 °C, the untreated specimen began to soften and deform. At 90 °C, it fractured completely while the furfurylatedone remained stable. This study demonstrated that furfurylation can improve wood properties and elongate its service life.

## 1. Introduction

With the increasing attention to environmental protection, sustainability, and biodiversity, natural high-quality forests are restricted to be traded all over the world, even not allowed to be harvested in most regions. Therefore, natural wood resources are decreasing, while the demand for wood products is increasing. In this context, people are driven to focus on the utilization of fast-growing plantation forests. However, most of the wood products from the plantation forest are poor in properties such as dimensional stability and mechanical strength, thus challenges exist during their service. To solve this problem, wood modification and functionalization techniques are widely used. Common wood modification methods include resin impregnation, among which urea-formaldehyde, phenolic-formaldehyde, and melamine-formaldehyde resins are the three most commonly used modifying agents [1]. Although these methods can significantly improve such properties of wood as dimensional stability, durability, and mechanical strength [2], the precursors contain environmentally unfriendly substances such as formaldehyde, making the fabrication and utilization of modified timber products a threat to the environment and human beings [2]. Recently, some advanced methods have been developed to reduce free formaldehyde, including the use of modified adhesives, formaldehyde scavengers, or alternative bio-based adhesives. Nevertheless, they all have relatively low bonding strength, poor water resistance, and other shortcomings [3].

As such, furfurylation stands out. The precursor of furfuryl alcohol (FA) is sustainable and renewable. FA is obtained from the processing residues of agricultural crops such as corn and wheat, which also makes it low-cost and readily available [4,5]. Secondly, as a substance with low molecular weight, low viscosity, and good water solubility, FA can be polymerized under normal or high temperatures with the presence of a catalyst [6]. Thirdly, the product is disposal friendly, since the disposal waste has almost minimum toxicity to the environment and human beings [4]. Furfurylated wood products are cost-effective and have excellent performance. In the long run, furfurylation also plays a role in improving the environment, reducing emissions of greenhouse gas, and promoting economic development [7].

Due to its unique advantages, furfurylation has gradually become one of the research hotspots in the field of wood modification in recent years. Nevertheless, there is no consistent conclusion on the mechanism of furfurylation. Two recognized reaction modes are available based on previous studies. Crosslinking reaction with wood cell wall components. This refers to the chemical grafting of FA to cell wall components (mainly lignin), so as to improve the dimensional stability of wood upon usage [8]. Nordstierna et al. stated that the furan polymer grafted to lignin via different NMR spectroscopic techniques, which proved the cross-linking reaction between FA and lignin [9]. The other mode is in situ polycondensation. FA was proven to be able to self-polymerize within wood cells at certain temperatures, which affects the hydrophobic properties and hardness of wood [10]. Meanwhile, the absence of new peaks in the NMR spectrum suggests that only self-polymerization occurred within wood [8]. However, in the currently reported wood furfurylation technology, the reaction mechanism between FA monomers and wood cell wall components is still unclear. This key issue makes it difficult to study the role and influence of the three main components of the wood cell wall during furfurylation.

Therefore, the objective of this study was to use alternative analytical methods to explore the reaction mechanism between FA and wood cell wall components from the perspectives of thermodynamics and microstructure change. The Douglas fir specimens were treated with furfuryl alcohol. The structural changes and the spatial distribution of FA in wood were revealed by visual characterization, and the thermodynamic parameters were evaluated by thermodynamic analysis technology. The reaction mechanism was proposed based on the results obtained from the above-mentioned characterization.

## 2. Materials and Methods

Raw material preparation and wood furfurylation

Douglas fir (*Pseudotsuga menziesii* (Mirb.) Franco) sapwood was selected as the raw material, and the specimens were cut into 10 mm × 10 mm × (5–10 mm) (R × T × L) samples that were oven-dried at 103 °C, then weighed and labeled according to the treatment formulation. Maleic anhydride (2 wt%, AR) was used as the catalyst, and sodium tetraborate (2 wt%, AR) as the stabilizer, both were supplied by Shanghai Macklin Biochemical Co., Ltd. (Shanghai, China), and used as received without further purification. The formulation of the solution is shown in Table 1. The FA solutions (30, 40, and 50 wt%, CP) turn to light yellow after settling down.

**Figure 1:**
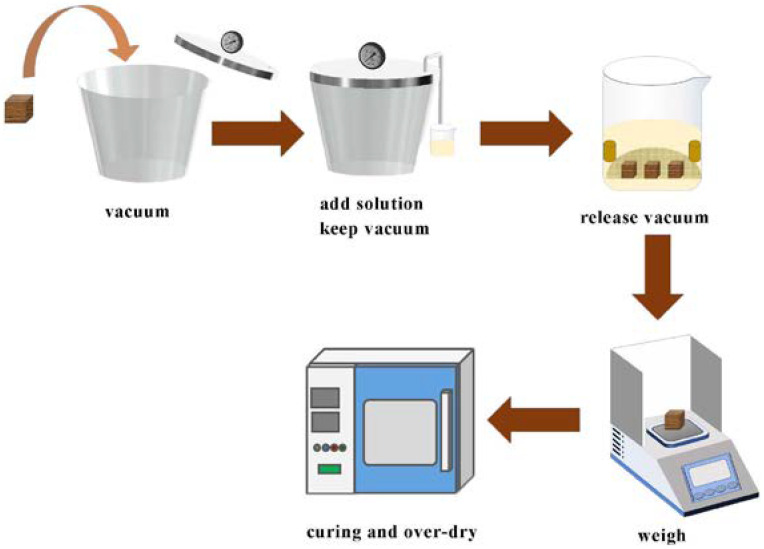
Diagram of the furfurylation process.

Furfurylation process (Figure 1): The specimens were subjected to a 30 min vacuum in the impregnation tank, after which the prepared solution was drawn into the tank with a hose under vacuum. The specimens were completely immersed in the solution. The vacuum was maintained after immersion for 1 h, then the vacuum was released, and the specimens were left in the solution for another 2 h. Finally, the impregnated specimens were wiped clean and weighed before being wrapped in aluminum foil and cured at 103 °C for 6 h. After curing, the aluminum foil was removed, and the specimens were dried at an increasing temperature gradient of 60 °C, 80 °C, and 103 °C for 4 h, respectively. The specimens were weighed and this value was recorded as the oven-dry weight. Ten replicates were used for each treatment.

### 2.1. The Weight Percent Gain (WPG) Measurement

The weight percent gain (WPG) is one of the important indexes to evaluate the polymerization degree of FA in wood. The high WPG value indicates that fewer FA molecules are volatilized without curing, and the polymerization degree of FA in the wood is high [1]. The WPG was calculated according to Equation (1):

(1) $$WPG=\frac{W_{1}-W_{0}}{W_{0}}\times 100\%$$

where *w*_0_ and *w*_1_ were the oven-dry weights of the specimen before and after impregnation. The ANOVA test was adopted to evaluate the statistical significance of FA concentration on the WPG.

### 2.2. Microstructural Visualization

#### 2.2.1. Fluorescence Microscopy (FM)

The specimens were microtomed into 20 μm thick (along the tree growth direction) slices and stained with toluidine blue. The distributions of lignin and polyfurfuryl alcohol (PFA) of the control and specimens treated with three FA concentrations were obtained using fluorescence microscopy (BX51, OLYMPUS, Tokyo, Japan). The exposure time was 350 ms and the contrast was set to 128.

#### 2.2.2. Scanning Electron Microscopy (SEM)

The control and specimens treated with three FA concentrations were observed using scanning electron microscopy (Phenom XL G2, Thermo Fisher Scientific, Waltham, MA, USA). 500× and 1500× magnification, 10 KV accelerating voltage, and BSD Full detector were used. Image J (2.9.0, National Institutes of Health, Bethesda, MD, USA) was used to compare and analyze the changes in cell wall thickness. Approximately 100 cells were selected in earlywood and latewood areas, respectively, to measure the wall thickness, average, and variance were calculated. The ANOVA test was adopted to evaluate the statistical significance of FA concentration on the cell wall thickness swelling.

#### 2.2.3. Confocal Raman Microscopy (CRM)

The control and furfurylated specimens were microtomed into 10 μm thick slices that were observed using a Renishaw inVia model confocal Raman microscopy (inVia, Renishaw, UK). A 532 nm laser was used for excitation. Raman spectra were collected using a CCD camera with a 300-lines mm^−1^ grating. 10 cycles of Raman spectra were recorded for each analysis point using a confocal aperture of 300 um, and each cycle was integrated for 0.5 s. Raman shifts were recorded and peaks were correlated to specific functional groups. Trials of Raman images for distinct distribution of cell wall components and PFA were attempted yet failed due to significant background fluorescence from chromophores.

### 2.3. Thermal Properties Characterization

#### 2.3.1. Differential Scanning Calorimetry Analysis (DSC)

The thermodynamic properties were analyzed using a NETZSCH DSC 204 F1 differential scanning calorimeter (Netzsch, Selb, Germany). The control and furfurylated specimens treated with three FA concentrations were ground into powders that could pass 80-mesh sieves. Samples were heated in a nitrogen gas (60.0 mL/min) from 0 °C to 160 °C at a heating rate of 5 °C min^−1^, kept isothermally at 160 °C for 5 min, and were then quenched to 30 °C to eliminate the thermal history. Next, the DSC thermograms were recorded by increasing the temperature to 160 °C at the same heating rate. Three replicates were tested for each group.

#### 2.3.2. Thermogravimetric Analysis (TGA)

The pyrolysis properties were analyzed using a NETZSCH TG 209 F3 thermogravimetric analyzer (Netzsch, Selb, Germany). The control and furfurylated specimens treated with three FA concentrations were ground into powders that could pass 80-mesh sieves. Samples were heated in a nitrogen gas (60.0 mL/min) from 25 °C to 700 °C at a heating rate of 20 °C min^−1^ and were then cooled to 25 °C at a cooling rate of 30 °C min^−1^. Three replicates were tested for each group.

#### 2.3.3. Dynamic Mechanical Analysis (DMA)

Tensile properties were analyzed using a NETZSCH dynamic mechanical analyzer (Netzsch, Selb, Germany). The control and furfurylated specimens treated with three FA concentrations were cut into a size of 0.1 mm × 5 mm × 10 mm (R × T × L). Samples were heated from 5 °C to 120 °C at a heating rate of 1 °C min^−1^ in tension mode, and the measurement frequency was set to 1 Hz. Three replicates were tested for each group.

## 3. Results

### 3.1. WPG Analysis

**Figure 2:**
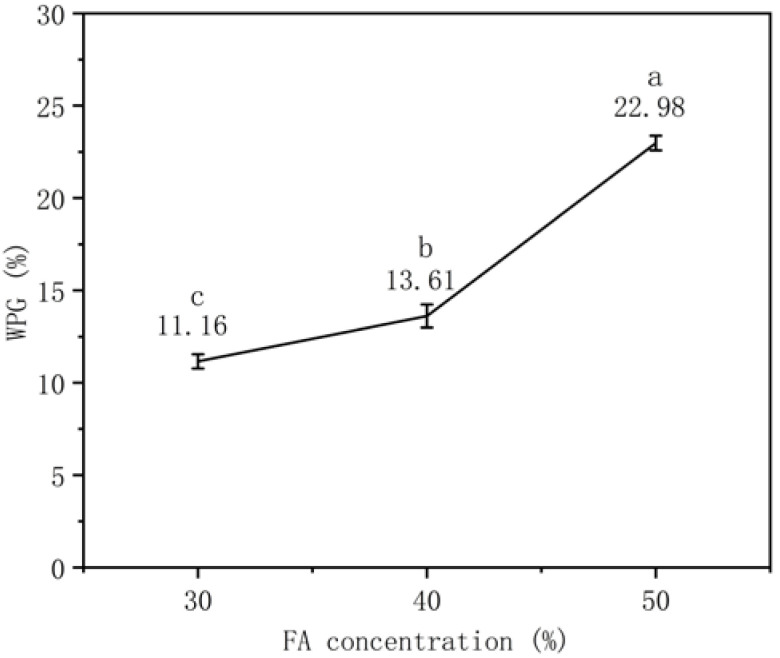
WPG (numbers) and their significant differences (letters) of specimens at different concentrations (30, 40 and 50%).

As shown in Figure 2, the WPG of the furfurylated specimens increases from 11.15% to 22.97% with the increase in the FA concentration from 30% to 50%. All data show significant differences (*p* < 0.05). When the concentration of FA continues to increase, as reported by other studies, the WPG will reach a peak at a certain point and then gradually decrease. Increasing FA concentration could lead to increased WPG, but some of the pits are occluded, preventing further impregnation [1]. In addition, the increase in WPG is also conducive to the improvement of dimensional stability, and durability [6]. Therefore, by changing the concentration of FA, WPG can be manipulated to achieve the ideal degree of modification.

### 3.2. Microstructural Characterization

**Figure 3:**
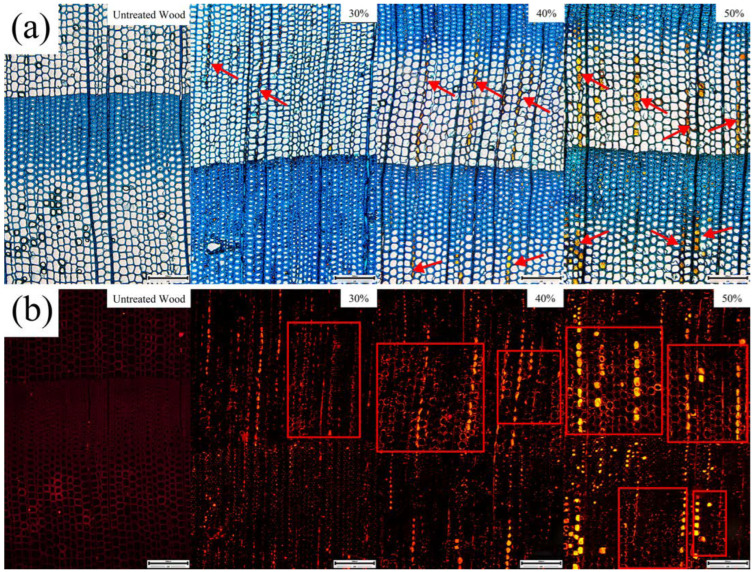
Ordinary (**a**) and fluorescent (**b**) micrographs before and after FA treatment. Red arrows and red squares are the PFA filling areas.

The optical and fluorescent micrographs of both control and furfurylated wood with different FA concentrations are shown in Figure 3. The structure of untreated Douglas fir was reported elsewhere, which was the same as shown in the present study [11]. Lignin autofluorescence was quenched by staining specimens with toluidine blue. Results showed that as the FA concentration increased, more polymerized FA (PFA) filled in the cell lumen, and the resultant furfurylated wood was brighter due to furan fluorescence, which coincided with the WPG trend of specimens treated with different FA concentrations. PFA was distributed radially in the tracheids and was mainly in earlywood at low FA concentration, then more PFA was found in latewood as the concentrations increased (Figure 3a). The reason for the PFA distribution trend was presumed that the tracheids in earlywood have thinner walls and larger lumen than that in latewood, providing wider routes for FA monomers and less vulnerability to embolism [12]. In addition to the fill-in of PFA in the cell lumen, the fluorescent graphs also generated brighter cell walls after furfurylation, indicating the penetration and polymerization of FA into the cell walls (Figure 3b).

Cross-section characterization via SEM

**Figure 4:**
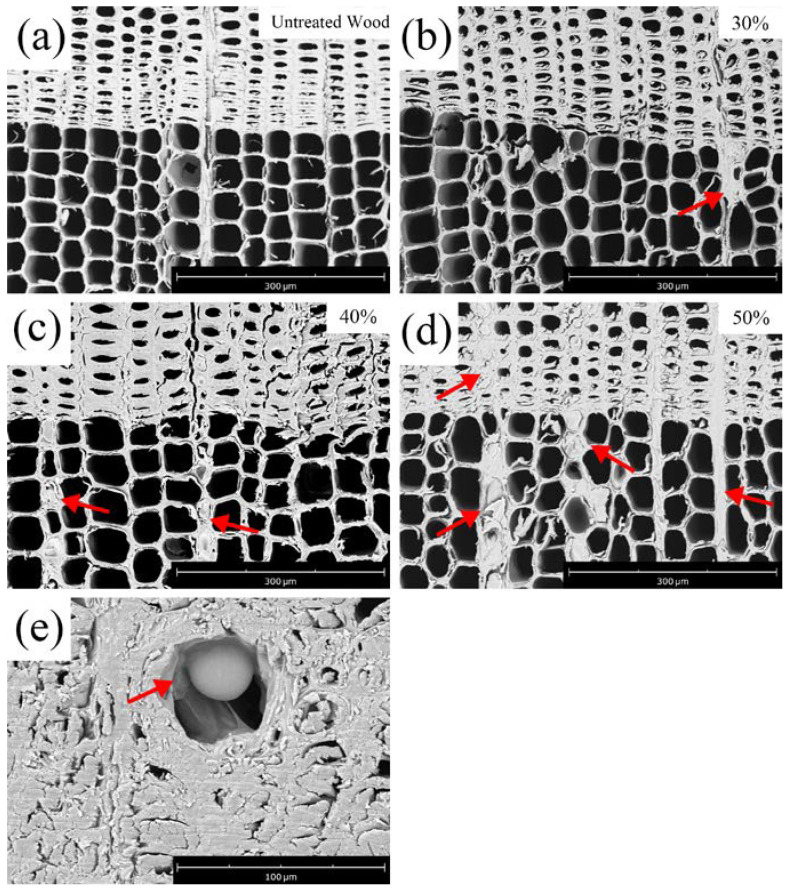
The cross-sectional structure of control wood (**a**), furfurylated wood (**b**–**d**), and resin canal (**e**) in SEM. The arrows show the deposition of PFA.

The cross-sectional structure of both control and furfurylated wood is presented in Figure 4. It is evident that FA was impregnated into the tracheids, rays, and resin canals.

Only a few cells were filled with PFA in the tracheids and rays for specimens treated with a 30% FA solution. A globular PFA was found deposited in the resin canal (Figure 4e) [13,14]. In addition, most of the furfurylated latewood cells had large deformation, which caused difficulty in the observation of cell wall thickness. After treatment with 40% FA, more tracheids and most of the ray parenchyma cells were covered with resin (Figure 4c). Therefore, it is proposed that FA may penetrate into cells through rays, tracheids, and resin canals. This is consistent with the study of Kong [15], who stated that modifiers could penetrate into wood through porous structures, including cell lumens and pits on cell walls. The filling range of PFA was greatly increased when treated with 50% FA, and the ray parenchyma cells were almost all filled with PFA (Figure 4d). It can be speculated that the ray cells were vital tissues for the transportation of modifiers into the wood.

**Figure 5:**
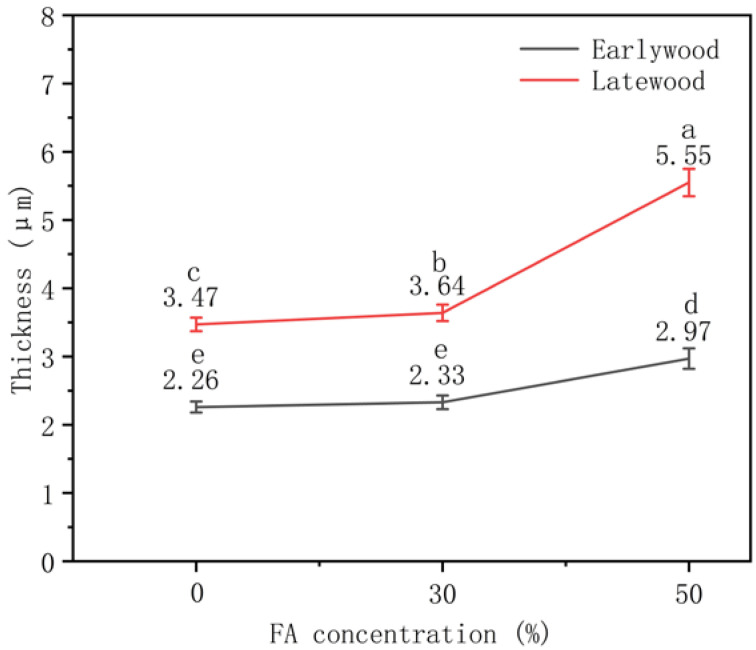
Cell wall thickness (numbers) and their significant differences (letters) of the control and furfurylated wood.

In addition to the changes in PFA distribution correlated to the FA concentration, the cell wall thickness also changed significantly. Image J was used to measure the thickness of cell walls. As shown in Figure 5, the average wall thickness of the control was 2.26 μm with a coefficient of variation (COV) of 15.8% for earlywood and 3.47 μm with a COV of 17.0% for latewood. The thickening of cell walls was not significant when treated with 30% FA, with only 3.10% and 4.81% thickening for the earlywood and latewood cell walls, respectively. In contrast, a significant thickening was achieved for those treated with 50% FA, with a cell wall thickening of 31.42% and 59.71%, respectively. Except for the earlywood of the untreated sample and furfurylated sample treated with 30% FA, other data showed statistical significance (*p* < 0.05). It was postulated that with the increase in FA concentration, the FA not only filled in the cell lumens but also polymerized in cell walls and become PFA. It diffused into the cell walls, causing cell walls to swell [8]. The penetrated PFA also formed a barrier within the wood that could prevent water absorption, resulting in a reduction in dimensional change in modified wood under various moisture contents [16].

**Figure 6:**
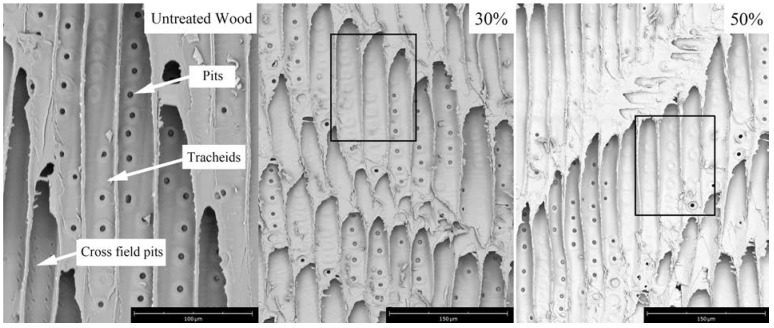
The variation in bordered pits status of the control and furfurylated specimens (radial-sectional structure). Black squares are pits filled with PFA.

The SEM graphs of the specimens in the radial section are shown in Figure 6. Bordered pits were not aspirated in the control wood [17], while some pits were filled with PFA (marked with a rectangular frame) for those treated with 30% FA. The results indicated that pits provided channels for FA penetration into cells. Most of the pits were filled with PFA after treatment with 50% FA.

Raman microscopy analysis

**Figure 7:**
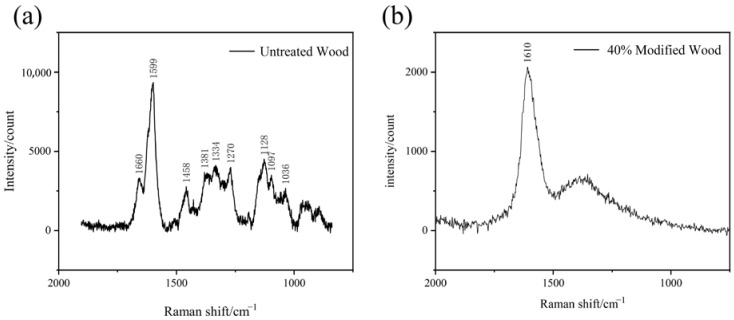
Raman spectrum of tracheid wall before FA treatment (**a**) and after FA treatment (**b**). Background fluorescence was obvious in furfurylated sample (**b**). The numbers in the figure are the characteristic peak values of Raman spectrum.

The control and furfurylated wood treated with 40% FA were characterized with Raman microscopy. The significant peak around 1599 cm^−1^ in the control wood was ascribed to the symmetric stretching vibration of the lignin benzene ring (Figure 7a). The peak at 1660 cm^−1^ was attributed to the coniferyl alcohol and coniferyl aldehyde structural units, which is another important characteristic peak of lignin. In addition, the characteristic peak around 1334 cm^−1^ was attributed to the bending vibration of the aliphatic hydroxyl of lignin. The one around 1270 cm^−1^ was attributed to the vibration of the hydroxyl or methoxy on the benzene ring of lignin or the ring vibration of the guaiacyl group. The characteristic peaks around 1122 cm^−1^ and 1095 cm^−1^ were attributed to the stretching vibration of the heavy atoms (C-C and C-O) of cellulose, xylose, and glucomannan [18,19].

However, strong fluorescence was found in the spectrum after furfurylation with 40% FA (Figure 7b), and only two characteristic peaks were evident at around 1610 cm^−1^ and 1350 cm^−1^. A further study using UV excitation will be tried to alleviate the background fluorescence [20].

### 3.3. Thermal Analysis

**Figure 8:**
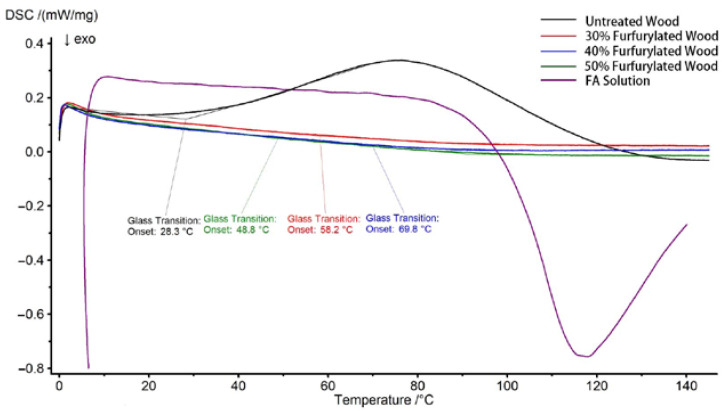
DSC curves of untreated and furfurylated specimens at different concentrations and FA solution.

DSC analysis of untreated and furfurylated specimens with different FA concentrations were given in Figure 8. There was a broad endothermic peak at around 76 °C for untreated wood samples, which might be due to the enthalpy relaxation of lignin at the glass transition, as the material gained entropic freedom [21,22,23]. In contrast, the trends of the DSC curves of the three groups of furfurylated wood were similar, without any endothermic or exothermic peaks within the programmed temperature range. This implied that the furfurylated specimens were completely cured; new stable chemical bonds were formed between lignin and FA, which had no enthalpy relaxation; and the thermal stability of modified wood was much better than the control. The glass transition temperature (T_g_) of all groups of specimens is listed in Table 2. The T_g_ values were calculated and generated by NETZSCH-Proteus-80 software. Results showed that the T_g_ increased with increased FA concentration. It was proposed that the rigid PFA formed both in the cell lumens and within the cell walls and the cross-linking reaction between FA and the cell wall components synergistically contributed to the increase in T_g_ [24,25] since either self-polymerization of FA or cross-linking reaction would result in thermosetting polymers with high rigidity [25]. A higher concentration of FA led to higher degrees of cross-linking reactions, which reduced the mobility of molecular chain segments of cell walls and finally had a higher T_g_. On the other hand, more reaction sites also formed for the self-polymerization of FA, which increased the T_g_.

In order to confirm the thermodynamic effect of FA on wood, the DSC analysis of 50% FA solution was conducted with the same temperature program. There was a significant exothermic peak starting from 90 °C, that reached the apex at around 116 °C, which is ascribed to the FA self-polymerization generating a large amount of heat [26,27]. Once the FA was impregnated into the wood and cured, no more exothermic peaks were found in the furfurylated wood, which again, indicated a thorough polymerization of FA monomers.

**Figure 9:**
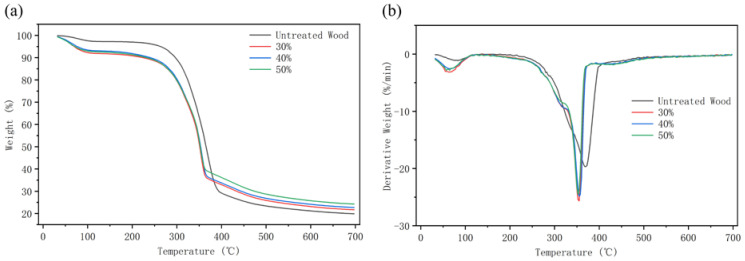
TG (**a**) and DTG (**b**) curves of untreated and furfurylated wood.

The thermal stability of wood before and after furfurylation was compared using TG (Figure 9a) and DTG (Figure 9b). Pyrolysis weight loss is summarized in Table 3. All groups of specimens underwent similar thermal degradation with slightly different loss rates. The TG curves showed that there is a slight weight loss under 100 °C and a sharp weight loss between 300 and 400 °C. Moreover, the derivative of the TG curve unveiled different degradation behaviors between the control and furfurylated wood in the temperature range of 300–400 °C.

The degree of thermal degradation under 100 °C was low, mainly due to moisture evaporation. However, the furfurylated specimens showed more weight loss than the untreated control, probably due to the chain scission and breakdown of unstable ether bonds, then evaporation of newly formed free formaldehyde [28].

Aggressive degradation occurred between 250 and 400 °C for all specimens. Studies have shown that the thermal degradation temperatures of cellulose, hemicellulose, and lignin are 315–400 °C, 220–315 °C, and 150–900 °C, respectively [29,30]. The thermal decomposition temperature of PFA is 160–480 °C, which is characterized by the breakdown of the carbon-oxygen bond in the furan ring [31].

The degradation peak for the untreated specimen was normally distributed, with the maximum degradation rate at 370 °C, where most of the holocellulose was degraded, and the lignin was also under severe decomposition. Nevertheless, for the furfurylated specimens, a shoulder was found at 325 °C. It was proposed that the integrity of the furan ring structure of PFA was compromised at this temperature range, and decomposed into ketonic moieties and enols [32]. The furfurylated specimens reached the highest degradation rate at 355 °C, slightly lower than the untreated ones. This might indirectly prove the cross-linking reactions between FA and cell wall components, and the newly formed covalent bonds were slightly more prone to be cleaved than that in the original cell wall components [9]. Moreover, there was an additional weak peak at 430 °C for the furfurylated specimens, which was ascribed to the further decomposition of PFA [32].

The decomposition rate slowed down above 450 °C, with only lignin degradation and graphitization thereafter [33,34]. The final mass loss percentage of the untreated specimen was 80.11%, while those of the furfurylated ones were 78.15%, 77.01%, and 75.68%, respectively, as shown in Table 3. The mass loss gradually decreased with the increase in FA concentration. The percentage mass residue of untreated, 30%, 40% and 50% specimens were 19.88%, 21.67%, 22.70% and 24.26%, respectively. The reason that furfurylated wood had a higher amount of residual carbon was that, in essence, there is higher carbon content in FA [10]. FA had a chemical cross-linking reaction with cell wall components and generated a new bond while rearranging and cyclizing aromatization by itself. It protected the wood to some extent, consequently, enhanced its thermal stability [35,36].

**Figure 10:**
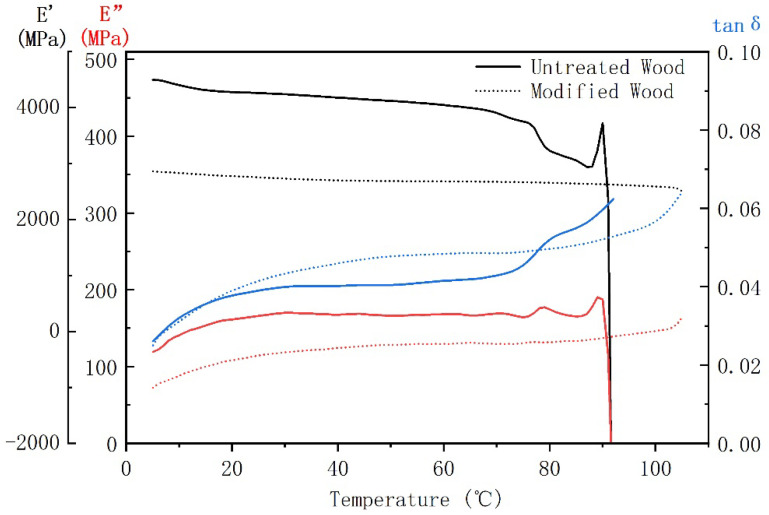
E′ (black line), E″ (red line) and tanδ (blue line) curves of untreated and furfurylated wood.

The thermo-mechanical behavior of untreated and furfurylated wood with 50% FA concentration was evaluated by DMA. The results of storage modulus (E’), loss modulus (E”), and damping factor (tanδ) are shown in Figure 10. Both the E’ and E” of the specimens decreased with increasing temperature, which was due to the fact that the mobility of the polymer chains in the cell wall increased with rising temperatures [37]. The lower E’ of the furfurylated wood indicated that the penetration and cross-linking effect of FA into the cell wall interrupted the crystalline structure of cellulose. Besides, the addition of PFA decreased the storage modulus due to intermolecular and intramolecular interactions that can alter the physical state of the wood matrix [32,36,38]. The E” of the untreated wood sample was always higher than that of the modified sample before its fracture, indicating that the incorporation of FA reduced the viscosity and the ability of energy consumption within the wood. Before the fracture occurred, the tanδ of the modified sample was higher than that of the untreated one, indicating that the molecules were less mobile and the wood dissipated more energy during the dynamic mechanical test. The reason was that the movement of the cellulose molecular chain was limited by FA and made the wood more brittle [36]. In addition, as the temperature reached about 75 °C, the untreated sample started to soften and deform. At 90 °C, it fractured completely while the modified one remained stable. This was in agreement with the DSC results, indicating that the incorporation of PFA into the wood matrix enhanced the ability of wood to resist deformation.

## 4. Conclusions

In this study, Douglas fir was modified with different concentrations of FA solution. The WPG, microstructure, and thermodynamic analysis were performed. The ANOVA results revealed that the WPG of wood increased significantly with increased FA concentration. FM and SEM results showed that the PFA was distributed in the tracheids, rays, and resin canals. With a higher FA concentration, a more uniform distribution of PFA was obtained. In addition, the cell walls were significantly thickened after furfurylation, with the swelling percentage reaching up to 59.71% upon 50% FA treatment. Pits provided channels for FA penetration into the cells. Moreover, compared with the control, the glass transition temperature of specimens increased, and the final mass loss percentage of the furfurylated wood decreased after furfurylation. Thermo-mechanical analysis indicated an improvement in thermal stability as well, due to the cross-linking of FA with cell wall components and the colonization of PFA in the cell lumens and walls. Therefore, furfurylation is an effective method for improving the quality of wood and the utilization of wood products.

## Figures and Tables

**Table 1:** Formulation of furfuryl alcohol (FA) impregnation solution.

FA (wt%)	Maleic Anhydride (wt%)	Sodium Tetraborate (wt%)	Deionized Water
30	2	2	66
40	2	2	56
50	2	2	46

**Table 2:** Glass transitions at different concentrations.

Concentrations of FA/%	Tg/°C
Untreated wood	28.3
30	48.8
40	58.2
50	69.8

**Table 3:** Pyrolysis weight loss of untreated and furfurylated wood at different temperature stages.

Temperature/°C	Pyrolysis Weight Loss Percentage/%			
	Untreated Wood	30% FA	40% FA	50% FA
25–250 °C	4.14	11.24	10.36	11.09
250–365 °C	45.50	52.22	51.81	49.36
365–700 °C	30.47	14.69	14.84	15.23
25–700 °C	80.11	78.15	77.01	75.68
